# Supplementary material for: Pregestational Prediabetes Induces Maternal Hypothalamic–Pituitary–Adrenal (HPA) Axis Dysregulation and Results in Adverse Foetal Outcomes
Source: Int J Mol Sci. 2024 May 16;25(10):5431. doi: 10.3390/ijms25105431 (PMC11122116; doi:10.3390/ijms25105431)
Supplement: Supplementary file 1 [file ijms-25-05431-s001.zip › ijms-2959635-supplementary.pdf]

3.10 Pups Systolic Blood Pressure

The systolic blood pressure of pups born from the non-prediabetic (NDP) female group (n=6 per group) and pups born from the prediabetic (PD) female group (n=6 per group) at week 3, 6 and 16. The systolic blood pressure in the pups born from the PD group was significantly higher when compared to pups born from the NPD group in all the experimental weeks.

Table S1: Systolic Blood Pressure in the pups born from the non-pre-diabetic (NPD) female and pre-diabetic (PD) female group (n = 6, per group) at week 3, 6 and 16.

**Groups (n=6)**		**Systolic pressure (mmHg)**
Week 3	NDP	119.5 ± 0.2305
	PD	122.2 ± 0.2072 ⋆⋆⋆
Week 6	NDP	119.7 ± 0.2029
	PD	122.4 ± 0.2633⋆⋆⋆
Week 16	NDP	120.6 ± 0.3646
	PD	128.7 ± 1.302 ⋆⋆⋆

Values are expressed as mean ± SEM. ⋆⋆⋆$p < 0.001$ denotes comparison with NPD.
